# PD-1 Blockade and TLR7 Activation Lack Therapeutic Benefit in Chronic Simian Immunodeficiency Virus-Infected Macaques on Antiretroviral Therapy

**DOI:** 10.1128/AAC.01163-19

**Published:** 2019-10-22

**Authors:** Elena Bekerman, Joseph Hesselgesser, Brian Carr, Mark Nagel, Magdeleine Hung, Adele Wang, Lance Stapleton, Agneta von Gegerfelt, Hanne Andersen Elyard, Jeffrey D. Lifson, Romas Geleziunas

**Affiliations:** a Gilead Sciences, Inc., Foster City, California, USA; b Bioqual, Inc., Rockville, Maryland, USA; c AIDS and Cancer Virus Program, Frederick National Laboratory for Cancer Research, Frederick, Maryland, USA

**Keywords:** human immunodeficiency virus, immunotherapy, simian immunodeficiency virus, Toll-like receptors

## Abstract

Antiretroviral therapy (ART) limits human immunodeficiency virus 1 (HIV-1) replication but does not eliminate the long-lived reservoir established shortly after viral acquisition. A successful HIV cure intervention necessitates either elimination or generation of long-term immune control of the persistent viral reservoir. Immune modulating strategies in conjunction with ART hold promise for achieving cure by inducing viral antigen expression and augmenting infected cell killing. Programmed death-1 (PD-1) blockade is a potential means to both activate and eliminate the latent reservoir by restoring exhausted T cell function. We assessed the therapeutic efficacy of PD-1 blockade, Toll-like receptor 7 (TLR7) activation with the agonist vesatolimod, or a combination of the two agents in chronically simian immunodeficiency virus (SIV)-infected macaques suppressed with ART for more than 2 years. Despite achieving extended anti-PD-1 antibody plasma exposure and TLR7-dependent immune activation after multiple administrations, neither individual treatment nor the combination resulted in changes to viral rebound kinetics following ART interruption or reduction in the SIV reservoir size. Our data in the context of other reports demonstrating improved viral control upon PD-1 blockade suggest that its therapeutic utility may be restricted to specific experimental conditions or treatment times during viral pathogenesis.

## Abstract (precis)

Antiretroviral therapy (ART) limits human immunodeficiency virus 1 (HIV-1) replication but does not eliminate the long-lived reservoir established shortly after viral acquisition. A successful HIV cure intervention necessitates either elimination or generation of long-term immune control of the persistent viral reservoir. Immune modulating strategies in conjunction with ART hold promise for achieving cure by inducing viral antigen expression and augmenting infected cell killing.

## INTRODUCTION

Human immunodeficiency virus 1 (HIV-1) infection, which involves the integration of viral DNA into the host’s genome, can lead to the establishment of latency early in infection, primarily within the CD4 T cell compartment (1–3). Antiretroviral therapy (ART) can inhibit viral replication and progression to AIDS but is required for life since treatment interruption inevitably leads to viral recrudescence from replication competent virus that persists despite ART (4, 5). Achieving ART-free control or elimination of long-lived, latently infected T cells thus represent major goals in the HIV cure field (6).

Among ongoing HIV cure efforts is the pursuit of novel immune-based therapeutics to eliminate cells harboring replication competent virus (7). One such strategy, with demonstrated clinical efficacy in oncology is the augmentation of the host’s immune response through immune checkpoint blockade (8). A well-characterized checkpoint molecule upregulated on T cells following antigenic stimulation is the programmed death-1 (PD-1) receptor. Engagement of PD-1 by its ligands, PD-L1 and PD-L2, inhibits T cell proliferation, cytokine secretion, and/or cytolytic potential (9–11). This natural biological response serves to dampen potentially harmful immune overactivation. Earlier work in a murine model demonstrated a key role of PD-1 in mediating T cell dysfunction during chronic infection with lymphocytic choriomeningitis virus (12). Subsequent studies extended this observation to additional chronic viral infections, including infections with hepatitis C virus, hepatitis B virus, HIV, and simian immunodeficiency virus (SIV) (13–17).

Characterization of PD-1 expression on peripheral blood mononuclear cells (PBMCs) from chronically HIV-infected individuals established a positive correlation between PD-1 overexpression on virus-specific CD4 and CD8 T cells and plasma viral load and a negative correlation between PD-1 and CD4 T cell counts (16). Moreover, *ex vivo* treatment of these PBMCs with an anti-PD-L1 antibody expanded the fraction of HIV-specific CD8 T cells and augmented production of IFN-γ upon peptide stimulation (16). Others demonstrated that CD4 T cells expressing PD-1, along with additional checkpoint molecules, T-cell immunoreceptor with Ig and ITIM domains (TIGIT) and lymphocyte-activation gene 3 (LAG-3), contribute to HIV persistence during ART (18). PD-L1 was also shown to be upregulated on antigen-presenting cells during HIV infection and served as a surrogate marker of disease progression (19).

Several preclinical studies have explored the utility of PD-1/PD-L1 blockade *in vivo* in SIV-infected macaques. Velu et al. demonstrated reinvigoration of SIV-specific cellular and humoral responses, reduced plasma viremia, and improved survival in chronically infected animals (predominantly late chronic) in the absence of ART (20). Other groups reported more limited and varied therapeutic benefit upon PD-1 axis blockade in conjunction with ART therapy, but the results supported further concept exploration under different experimental conditions or in combination with other agents (21–23).

Toll-like receptor 7 (TLR7) is an innate immune pattern recognition receptor, whose ligands are single-stranded and short double-stranded RNAs. TLR7 engagement stimulates antiviral immunity by triggering dendritic cell maturation, cytokine secretion, and antigen presentation and in turn enhances adaptive immune responses (24). Potent and selective TLR7 agonists, such as vesatolimod (VES; GS-9620) have been shown to (i) modestly induce HIV production from infected PBMCs *ex vivo*, (ii) activate T cells, and (iii) enhance antibody-mediated killing of HIV^+^ CD4 T cells *in vitro* (25). Several reports have recently uncovered a potential for orally delivered TLR7 agonists to induce viral control in a subset of SIV- or chimeric simian/human immunodeficiency virus (SHIV)-infected macaques, either alone (26) or in combination with a therapeutic vaccine or a broadly neutralizing anti-envelope antibody (27, 28).

To date, most HIV cure strategies have been tested in preclinical models or clinically in conjunction with ART (29). In this study, we set out to characterize the therapeutic potential of a PD-1 blocking antibody alone or in combination with the TLR7 agonist vesatolimod in a chronically SIV-infected, long-term ART-suppressed rhesus macaque model. Our results demonstrate that the PD-1 blocking antibody alone or in combination with vesatolimod was well tolerated and yielded an expected pharmacodynamic outcome. However, neither agent alone nor the combination prevented or delayed viral rebound or induced delayed control of viremia after ART release in this model.

## RESULTS

### Human/rhesus chimeric anti-PD1 antibody.

**FIG 1:**
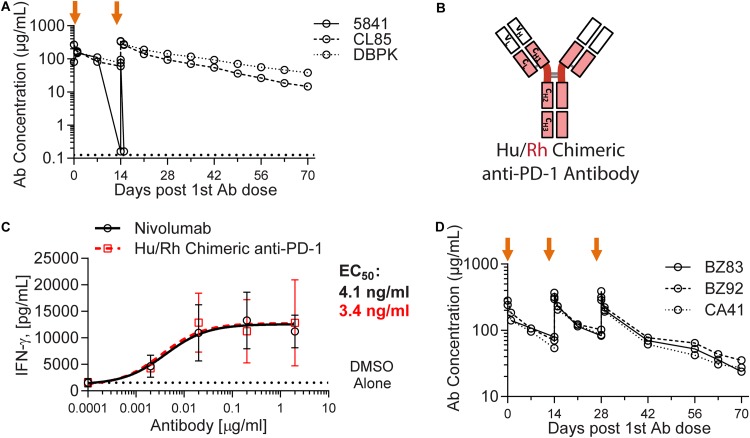
Human/rhesus chimeric anti-PD-1 antibody exhibits reduced immunogenicity in rhesus macaques. (A) Time course of plasma concentration of nivolumab (anti-PD-1 human antibody) following two i.v. administrations (orange arrows) at 10 mg/kg to three naive rhesus macaques. A sharp decrease in detectable anti-PD-1 antibody exposure in animal 5841 is indicative of anti-drug antibody (ADA) formation. The lower limit of quantification is 0.125 μg/ml. (B) Schematic representation of the chimeric human (hu)/rhesus (rh) antibody construct. (C) IFN-γ production in a CMV-recall response assay conducted in the presence of a dose-titration of nivolumab (black) or the chimeric antibody (red). Calculated EC_50_ values for each antibody are denoted in their corresponding color. (D) Time course of plasma concentration of the human/rhesus chimeric anti-PD-1 antibody following three i.v. administrations (orange arrows) at 10 mg/kg to three naive rhesus macaques.

Nivolumab (Opdivo; Bristol-Myers Squibb), an anti-PD-1 antibody approved for multiple oncology indications, was selected to functionally block the PD-1 receptor in our nonhuman primate study. After administration to naive rhesus macaques, the plasma exposure of this fully human anti-PD-1 antibody dropped sharply in one of three animals, a result indicative of immunogenicity and induction of anti-drug antibodies (ADA) (Fig. 1A). ADA has been previously described in cynomolgus macaques treated with nivolumab (30). To reduce nivolumab immunogenicity and to enable repeat dosing with extended exposure, we generated a chimeric antibody by replacing the nucleic acid sequence encoding the human antibody constant region with that of the rhesus macaque (Fig. 1B). To ensure that the modification did not impair functionality, the chimeric antibody activity was tested side by side with nivolumab in an assay that evaluates cytomegalovirus (CMV) recall responses (Fig. 1C). The potencies of the two antibodies were comparable in this assay (a 50% effective concentration [EC_50_] of 4.1 ng/ml for nivolumab versus 3.4 ng/ml for the chimeric antibody). The pharmacokinetic profile of the chimeric anti-PD-1 antibody was then assessed in a multiple-dose intravenous (i.v.) infusion study in three naive rhesus macaques, revealing no apparent exposure-limiting immunogenicity (Fig. 1D). This chimeric antibody was advanced for testing in SIV-infected, ART-suppressed rhesus macaques.

### Viral suppression and efficacy study design.

**FIG 2:**
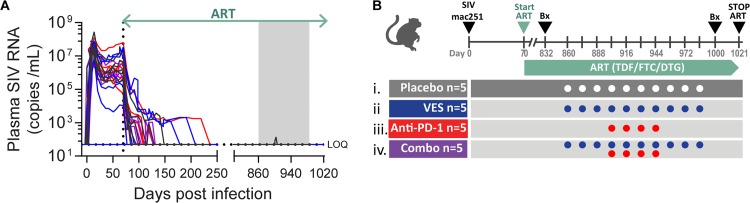
Efficacy study design and plasma viral loads during treatment. (A) Longitudinal measurements of plasma SIV RNA from the time of infection through ART release. The lower limit of quantification is 50 SIV RNA copies/ml. Individual animal colors correspond to the assigned treatment group specified in panel B. The box shaded in gray represents the therapeutic intervention with VES ± anti-PD-1 antibody. (B) Schematic of the efficacy study design denoting the time of infection with SIVmac251, the start and stop of ART therapy, the biopsy (Bx) time points, and the dosing of anti-PD-1 antibody and VES.

To assess whether the human/rhesus chimeric anti-PD-1 antibody and vesatolimod (VES) could cure an AIDS virus infection or confer ART-free virologic control, these agents were tested in SIV-infected, ART-suppressed rhesus macaques. To establish the animal cohort, 20 Indian-origin rhesus macaques, which did not express Mamu-A*01, -B*08, and -B*17 controller major histocompatibility complex alleles, were infected intrarectally with SIVmac251, and their plasma viral loads were tracked longitudinally (Fig. 2A). At 70 days after confirmed infection, corresponding to the early chronic set points ranging from 8 × 10^3^ to 4 × 10^7^ RNA copies/ml, the animals were placed on a daily subcutaneously administered ART formulation consisting of tenofovir disoproxil fumarate (TDF), emtricitabine (FTC), and dolutegravir (DTG). Plasma SIV RNA levels decreased to less than 50 copies/ml within 2 to 6 months and remained below this level until the therapeutic intervention at 26 months after ART initiation. The cohort was stratified into four treatment groups (*n* = 5/group), balancing animal weights, complete blood counts, and clinical chemistries, which were measured 2 weeks prior to the first VES treatment. The groups received (i) vehicle control, (ii) VES at 0.15 mg/kg by oral gavage, (iii) anti-PD-1 chimeric antibody at 10 mg/kg by i.v. infusion, or (iv) a combination of the two agents (Fig. 2B). VES was administered every other week for a total of 10 doses, while the anti-PD-1 antibody was administered every other week after three lead-in VES doses (in the combination group) for a total of four doses. Plasma viral loads remained below 50 SIV RNA copies/ml throughout the ART phase, including during VES and anti-PD-1 dosing with the exception of one measurement of 139 copies/ml in a single placebo control animal. Routine clinical observations, complete blood counts, and blood chemistry evaluations did not reveal any adverse events or tolerability issues with either agent or the combination (data not shown).

### TLR7 agonist (VES) pharmacokinetics and pharmacodynamics.

**FIG 3:**
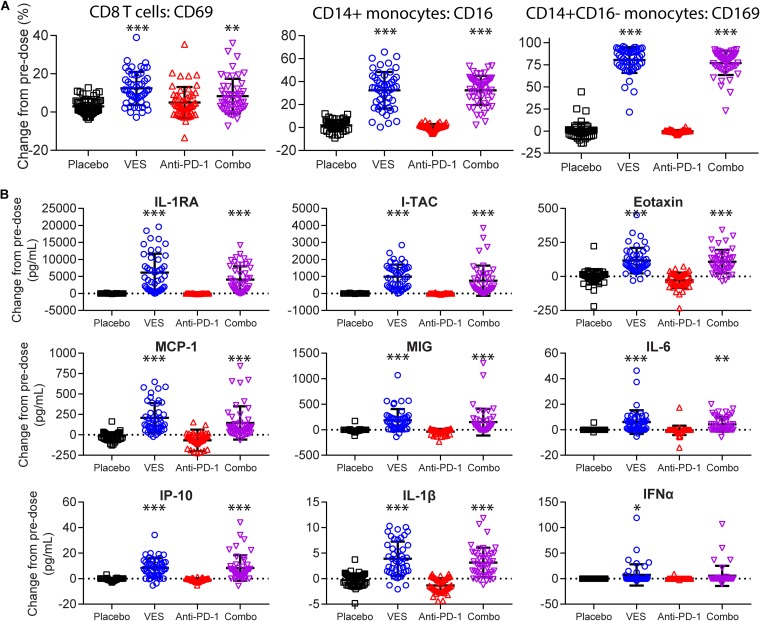
VES Pharmacodynamic response. (A) Percentage of CD69^+^ cells among peripheral CD8^+^ T cells (left), CD16^+^ cells among CD14^+^ monocytes (middle), and CD169^+^ cells among CD14^+^ CD16^−^ monocytes (right) assayed by flow cytometry and expressed as change from predose to 24 h postdose. (B) Expression of nine plasma cytokines and chemokines assayed by multiplex Luminex and expressed as the change in pg/ml from predose to 24 h postdose. Each symbol represents one animal at one time point, with all the time points grouped into a single column. Group averages and standard deviations are marked in black. ***, *P* < 0.001; **, *P* < 0.01; *, *P* < 0.05 (one-way ANOVA test with Dunnett multiple-comparison correction).

Given the rapid absorption and hepatic first-pass extraction of VES, detectable plasma concentrations were only measured at 30 min postdose (see Fig. S1 in the supplemental material) and were below the limit of detection in all animals at 24 h postdose. To assess the pharmacodynamic response to VES, we evaluated the peripheral immune cell activation status, as well as plasma cytokine levels at 24 h after each dose. We observed a statistically significant induction of the activation marker CD69 on the surfaces of CD8^+^ T cells upon dosing the animals with VES either alone (mean, 12.4% ± 8.6%) or in combination with the anti-PD-1 antibody (mean, 8.3% ± 9.1%) relative to the placebo control (mean, 3.0% ± 4.0%) or to anti-PD-1 antibody alone (mean, 5.0% ± 8.1%) (Fig. 3A). Similarly, we observed a trend in surface CD69 upregulation on CD4^+^ T cells and B cells from all treatment groups, although the increases did not reach statistical significance compared to the placebo (Fig. S2). On CD14^+^ circulating monocytes, we detected a 32% average increase from baseline of CD16 (FCγ-RIII receptor) expression in both VES and combination treatment groups, a finding indicative of monocyte differentiation into the nonclassical, proinflammatory subtype. There were only 0.7 and 1.8% average increases in the CD16 levels in anti-PD-1 and placebo groups, respectively (Fig. 3A) (31). Furthermore, VES administration, but not anti-PD-1 or placebo control, resulted in robust induction of CD169 (Siglec-1) marker among circulating CD14^+^ CD16^−^ monocytes (80 and 77% average increase from baseline in VES and combination groups versus 0% in placebo or anti-PD-1 groups) (Fig. 3A). Induction of CD169 on monocytes has previously been demonstrated in response to multiple TLR ligands in a type I interferon (IFN)-dependent manner and implicated in mediating immune cell-cell interactions (32). From a panel of diverse cytokines, we measured a significant induction of proinflammatory cytokines (interleukin-1β [IL-1β], IL-6, and IFN-α), chemokines (MCP-1/CCL2, MIG/CXCL9, I-TAC/CXCL11, and eotaxin/CCL11, IP-10), and IL-1 receptor antagonist (IL-1RA) in the VES-treated groups (Fig. 3B). The following proteins were also measured as part of a Luminex assay panel but were either not detected or not significantly changed relative to the placebo control group at the 24-h time point: IFN-γ, tumor necrosis factor alpha, IL-2, IL-4, IL-5, IL-8, IL-10, IL-12, IL-15, IL-17, MIP-1α, MIP-1β, RANTES, MIF, MDC/CCL22, granulocyte-macrophage colony-stimulating factor, and granulocyte colony-stimulating factor.

### Anti-PD-1 antibody pharmacokinetics and pharmacodynamics.

**FIG 4:**
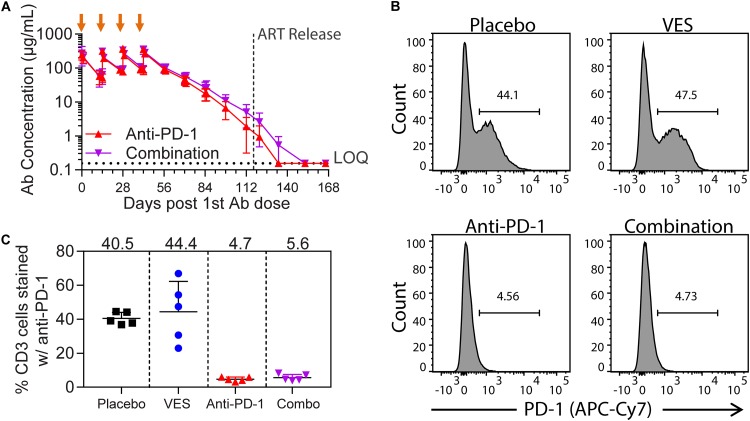
Anti-PD-1 antibody plasma exposure and receptor occupancy. (A) Time course of plasma concentration of the chimeric anti-PD-1 antibody after four i.v. administrations (orange arrows) at 10 mg/kg to five rhesus macaques per group. The average values and standard deviations per group are plotted. The lower limit of quantification is 0.156 μg/ml. (B) Percentage of peripheral CD3^+^ T cells staining positive by flow cytometry with an anti-PD-1 antibody cross-reactive with the epitope for nivolumab. The sample time point corresponds to 2 weeks after the first chimeric anti-PD-1 antibody dose. The loss of PD-1 positive signal is indicative of PD-1 receptor occupancy. A representative plot from each treatment group is shown. (C) Quantification of the percentage of CD3^+^ T cells staining positive with anti-PD-1 antibody, as demonstrated in panel B. Group means are noted at the top.

The chimeric anti-PD-1 antibody plasma exposure was confirmed after each dose and its elimination tracked for 19 weeks after the last administration to determine when levels declined below the limit of quantification (0.156 μg/ml). The anti-PD-1antibody half-life in rhesus macaques was 11.9 days. Similar to the healthy-animal study (Fig. 1D), the average *C*_max_ for the 10 SIV-infected ART-suppressed animals dosed with anti-PD-1 antibody ranged from 263 μg/ml on dose 1 to 354 μg/ml on dose 4, with no evidence of exposure-limiting immunogenicity (Fig. 4A). We further confirmed PD-1 receptor occupancy 2 weeks after the first anti-PD-1 antibody dose by flow cytometry. To do so, we stained PBMCs derived from study animals with a different anti-PD-1 antibody (clone NAT105) that competes for the same epitope and quantified detectable surface PD-1 levels (representative plots are shown in Fig. 4B). Although an average of 42% of CD3^+^ T cells had detectable surface PD-1 expression in the placebo control and the VES-treated groups, the staining in this population was reduced to below 6% in the anti-PD1 antibody-dosed groups, demonstrating significant receptor occupancy (Fig. 4C).

### ART discontinuation.

**FIG 5:**
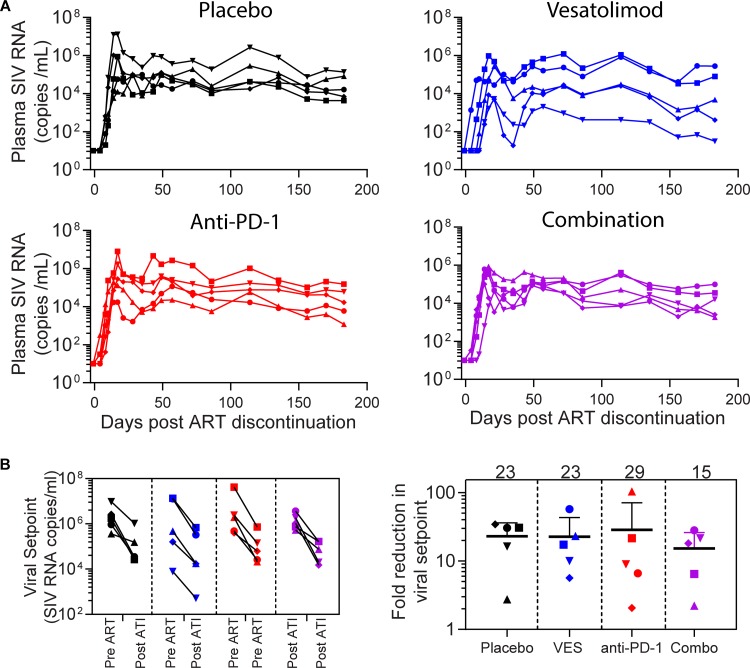
Viral rebound after ART discontinuation. (A) Plasma SIV viral loads from ART discontinuation to the end of the study. (B) Absolute values of SIV set points established pre- and post-ART discontinuation in individual animals (left). The fold reduction in set points is indicated on the right. Group means are noted at the top.

One month after the last administration of VES, ART was discontinued to monitor viral rebound kinetics. All 20 animals rebounded within 2 weeks of ART discontinuation despite several years of viral suppression. There was no significant delay in viral rebound with any of the treatments relative to placebo (Fig. 5A). Viral loads peaked 2 weeks after stopping ART and reached a set point, which ranged from 5 × 10^2^ to 1 × 10^6^ RNA copies/ml (Fig. 5B). Consistent with prior reports, post-ART viremia levels were reduced by over 1 log from pre-ART set points. However, the overall magnitude of the reduction was comparable across the four treatment groups (Fig. 5B). All animals remained viremic with no evidence of delayed virologic control through 6 months after stopping ART.

**FIG 6:**
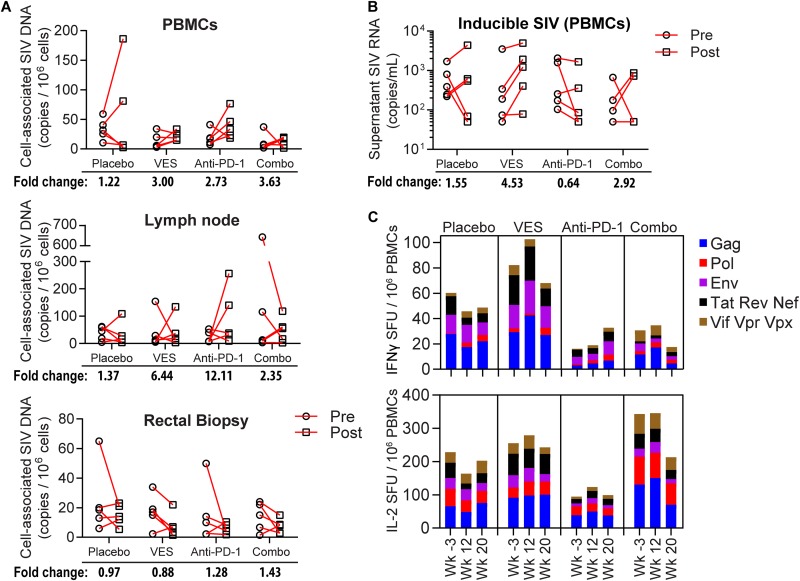
SIV DNA quantification and SIV-specific T cell responses pre- and posttherapy. (A) Cell-associated SIV DNA per million cells from PBMCs (top), lymph node biopsy (middle), and rectal pinch biopsy (bottom) assayed by qPCR. The average fold change from pre- to posttreatment course is noted at the bottom. (B) Inducible SIV quantified by qRT-PCR from the supernatants of PBMCs harvested from the animals pre- and posttherapy and induced with ConA *in vitro* for 6 days. The average fold change from pre- to posttreatment is noted at the bottom. (C) SIV-specific T cell responses measured by IFN-γ and IL-2 FluoroSpot ELISPOT assay after stimulation with SIV peptide pools. Weeks –3, 12, and 20 correspond to the pretreatment baseline, predose 7, and 2 weeks after dose 10, respectively.

Despite comparable virus rebound in all four arms of this study, we nonetheless evaluated the effect of PD-1 blockade and VES on cell-associated total viral DNA and *ex vivo* virus production. We first measured total cell-associated viral DNA (CAVD) by qPCR in PBMCs, as well as lymph node and rectal biopsy samples, taken just prior to the first VES dose and 2 weeks after the last dose but prior to ART discontinuation. Figure 6A, which shows the absolute change in CAVD copies per million cells from pre- to posttreatment time points, revealed no clear trend in the total reservoir dynamics. The average fold differences in CAVD were largely within 5-fold of pretreatment but fluctuated between an overall decrease and an increase in different animals within a group, potentially reflecting sampling variation. To measure the *ex vivo* inducible SIV reservoir, we stimulated PBMCs from study animals with a potent T cell mitogen, concanavalin A (ConA), and quantified SIV RNA in the cell supernatant 6 days later by qRT-PCR (see Fig. S3 in the supplemental material). Comparable to the CAVD measurement, we detected <5-fold increases or decreases in the inducible viral reservoir, on average, with no statistical difference between the treatment groups and placebo (Fig. 6B).

Lastly, we evaluated SIV-specific T cell responses prior to and after the anti-PD-1 and VES interventions while the animals were still on ART by multiparameter FluoroSpot ELISPOT (enzyme-linked immunosorbent spot) assay. A total of seven pools each comprising 100 to 160 peptides spanning the entire SIV proteome were used to stimulate PBMCs, and the resulting IFN-γ and IL-2 responses were quantified. Cumulative group ELISPOT responses revealed <100/million IFN-γ-producing cells and <400/million IL-2-producing cells at baseline (week −3; Fig. 6C). These responses were marginally augmented by the administration of VES and/or anti-PD1 antibody by dose 7 (week 12) but returned to or below baseline at the conclusion of the dosing phase (week 20). Collectively, these data revealed no impact on the viral reservoir or the frequency and function of SIV-specific T cells after PD-1 blockade or VES administration in this ART-suppressed model.

## DISCUSSION

Immune checkpoint inhibition is an approach being explored for HIV cure due to the potential to both activate the latent reservoir and enhance immune clearance of infected cells. To date, published evidence for the antiviral efficacy of PD-1 blockade in SIV-infected nonhuman primates has been demonstrated either in the absence of ART or when initiated in conjunction with ART (20, 33). In the present study, we investigated the therapeutic efficacy of PD-1 blockade with or without TLR7 activation in a nonhuman primate model that mimics conditions commonly found in the clinic: long-term virological suppression by ART. After 2.5 years of suppressive ART combined with a 5-month intervention consisting of ten TLR7 agonist administrations (VES) and four anti-PD-1 antibody administrations, ART was interrupted to assess whether these treatments could cure the monkeys of SIV or lead post-ART virologic control. Generation of the human/rhesus chimeric anti-PD-1 antibody allowed us to circumvent induction of exposure-limiting ADA in the rhesus macaques and to achieve sustained plasma exposures similar to those seen with the therapeutically active 3-mg/kg nivolumab treatment in humans (34). Repeat dosing with the chimeric anti-PD-1 antibody resulted in *C*_trough_ concentrations of >10 μg/ml for more than 3 months, similar to the steady-state trough concentrations attained with nivolumab treatment in humans. Repeat dosing with VES (every 2 weeks), characterized by rapid absorption and clearance (35), provided pulsatile immune activation over a 5-month period.

Despite having plasma viral loads of <50 RNA copies/ml for 20 to 24 months, viral recrudescence occurred in all treatment groups within 2 weeks of ART discontinuation. Likely as a result of prolonged ART therapy, we observed more than a log reduction in viral load set points when comparing post- to pre-ART time points. However, no further reduction was evident with the anti-PD1 and VES treatments relative to the ART-only placebo control. Similarly, cell-associated SIV DNA or *ex vivo* inducible SIV levels in tissue biopsy specimens or PBMCs were not affected by the treatments, compared to the ART-only placebo. Minor reservoir fluctuations measured in individual animals may represent variation in sampling of the infrequent SIV-infected cells in animals on long-term ART. The lack of effect of the anti-PD1 and VES treatments on these viral reservoir parameters is consistent with the lack of viral rebound phenotype during ART discontinuation.

Given the measurable plasma exposure and receptor occupancy by the anti-PD1 antibody, a lack of therapeutic efficacy may be explained at least in part by the potent ART suppression for over 2 years, which is known to reduce PD-1 expression and markedly reverse immune exhaustion (22, 36). In fact, administration of PD-1 blocking antibody after just 6 months of ART suppression resulted in less pronounced restoration of T cell function compared to PD-1 treatment of the same animals before ART initiation in a study by Mylvaganam et al. (33). Thus, the lack of an increase in SIV-specific functional cytotoxic T lymphocytes (CTLs) from our long-term ART suppressed macaques is consistent with a trend of reduced benefit of checkpoint blockade with increased time on ART. Moreover, it is plausible that the therapy improved functional CTL frequency within the sites harboring the bulk of SIV reservoir (i.e., the secondary lymphoid organs or the gut associated lymphoid tissue), but sampling from the periphery did not reflect this.

A recent report by Borducchi et al. demonstrated a lack of viral rebound after ART cessation and after treatment with a combination of TLR7 agonist plus a broadly neutralizing anti-envelope antibody (28). Although it may be concluded that the TLR7 activation and PD-1 blockade tested in our combination study was less effective at mediating reservoir clearance than a TLR7 agonist with a broadly neutralizing antibody, the two animal models were substantially different, which does not permit a fair comparison. The Borducchi et al. study relied on an acute 7-day infection model before starting ART, which better preserves the immune system and results in a relatively small viral reservoir. In addition, Borducchi et al. used a chimeric SIV/HIV strain, which leads to lower peak viremia and lower chronic set points, and this less-robust virus can occasionally be naturally cleared in subsets of animals. Our study tested the curative potential of simultaneous TLR7 activation and PD-1 blockade in animals that established early chronic infection before starting ART and used a more pathogenic virus, SIVmac251, which yields higher peak viremia and higher chronic set points and is rarely spontaneously cleared. Since early ART limits reservoir establishment (37), the bar for achieving cures or reservoir reductions in a chronic setting is considerably higher.

Since the anti-PD-1 antibody plasma levels were substantially below the therapeutic levels prior to ART discontinuation, we can only draw conclusions regarding the anti-PD-1 antibody’s latency reversal and curative potential under long-term ART but not its efficacy during the plasma viral rebound phase. A recent report by McGary et al. revealed that a memory CD4 T cell population contributing to SIV persistence during ART expressed CTL-associated antigen 4 (CTLA-4) but not PD-1; thus, dual inhibition may be required to successfully expose and eliminate the reservoir (38). The lack of transient plasma viremia, which was previously documented with TLR7 agonists administered to ART-suppressed rhesus macaques (<1 year aviremic [26]), also suggests that in our study VES did not induce robust reactivation of the reservoir, despite detectable immune cell activation, thus precluding elimination of the viral reservoir. In future studies, we aim to test other immune modulating agents and combinations with anti-envelope antibodies capable of recruiting effector cells to attempt to augment the therapeutic potential of VES in this model.

It is plausible that shorter duration of ART prior to anti-PD-1 and/or VES treatment could have facilitated the production of detectable SIV and enhanced infected cell clearance. However, if short-term ART suppression in conjunction with substantial immune cell exhaustion prior to treatment is required to reveal the potential efficacy of immune checkpoint blockade in HIV, it would inevitably limit the patient population for whom such therapy may provide clinical benefit. Higher VES doses resulting in greater pharmacology could improve immune activation in this model but are unlikely to be broadly tolerated in humans. Similarly, more frequent TLR7 agonist administration, which is subject to a tachyphylactic pharmacodynamic response, as shown by Bourquin et al. (39), with frequent administration of a TLR7/8 agonist (R848) in a murine model may have only limited utility. However, increasing the total numbers of TLR7 agonist doses might increase the efficacy.

Beyond the animal model, human clinical experience with PD-1 blockade in HIV-1 infection is limited largely to individuals treated for cancer comorbidities. A report on a single HIV-positive lung cancer patient treated with nivolumab demonstrated multilog viral reservoir reduction accompanied by transient viremia on ART and enhanced immunity (40). However, other reports of checkpoint blockade did not reveal any impact on measures of HIV persistence (41, 42). Notably, Scully et al. documented no consistent changes in HIV DNA or RNA, frequency, and responses of virus-specific T cells with anti-PD-1 therapy, even when a complete response was achieved in the primary oncology indication (42). A small phase I prospective study in HIV-infected ART-suppressed patients receiving a single low dose of anti-PD-L1 antibody (BMS-936559) revealed an apparent enhancement of HIV-specific responses in two of six participants but no accompanying change in HIV RNA or DNA (43). Although the trial was discontinued prematurely, this study hinted at the potential for a response in a subset of patients if extended dosing of an anti-PD-L1 antibody was tolerated. Unfortunately, understanding of biomarkers predictive of patients’ responsiveness to checkpoint blockade is still incomplete in oncology, and even more so in HIV given the limited clinical experience. Thus, characterization of immune and virologic correlates of response in future preclinical and clinical studies will be of critical importance.

Given the scope of potential immune-related adverse events associated with checkpoint inhibition (44), it is paramount that we understand the likely benefit of such interventions when treating otherwise healthy people living with HIV on successful ART regimens. Additional ongoing, preclinical and clinical evaluations of checkpoint inhibition will further our understanding of the value of this approach for HIV cure. Our study in the broader context of reported therapeutic benefit of PD-1 blockade in viremic animals or in conjunction with ART initiation suggests that the timing of therapy relative to infection and ART treatment may be a critical determinant of the efficacy potential (20, 33). This should inform the design of future studies.

## MATERIALS AND METHODS

### Animals.

Twenty outbred, Indian-origin, adult male rhesus macaques (Macaca mulatta) that did not express the class I controller alleles Mamu-A*01, Mamu-B*08, and Mamu-B*17 were housed and handled at Bioqual, Inc., Rockville, MD. Animals were infected via intrarectal challenge with the SIVmac251 strain as previously described (27).

The formulated ART cocktail (Gilead Sciences, Inc.) contained TDF (5.1 mg/ml), FTC (40 mg/ml), and DTG (2.5 mg/ml) and was administered subcutaneously once daily at 1 ml/kg (body weight) (28). Formulated VES (Gilead Sciences, Inc.) contained VES (0.15 mg/ml) and 0.005% (wt/vol) propyl gallate in water-HCl (pH 2 to 3). The formulated anti-PD-1 antibody (Gilead Sciences, Inc.) contained the antibody (10 mg/ml) in a buffer with 20 mM sodium citrate, 50 mM NaCl, 3% (wt/vol) mannitol, and 20 μM pentetic acid (pH 6). VES and the anti-PD-1 antibody were administered every other week under anesthesia. Macaques were bled at various time points to assess pharmacokinetics, pharmacodynamics, complete blood counts and blood chemistry, immunological parameters, viral loads, and intracellular viral reservoirs. Animals underwent two lymph node and rectal pinch biopsies for tissue viral reservoir measurements. PBMCs and biopsy specimens were cryopreserved for downstream viral reservoir quantification.

### Study approval.

All the procedures described were approved by the appropriate Institutional Animal Care and Use Committee at Bioqual, Inc.

### Human/rhesus chimeric anti-PD-1 antibody cloning and production.

Heavy- and light-chain sequences of nivolumab (US 20100266617A1), a monoclonal human IgG4 antibody with a kappa light chain, were contract synthesized by GeneArt (Thermo Fisher Scientific) and cloned into pcDNA3.1 vector. The variable heavy (VH) and light (VL) fragments of nivolumab were PCR amplified from the human antibody expression vectors and cloned into pcDNA3.1 vectors carrying the constant region sequences of rhesus IgG4 and kappa, respectively, to make the human/rhesus chimera. The constant heavy- and light-chain sequences of rhesus macaque antibody were amplified from pFUSE-CHIg-rhG4 and pFUSE2-CLIg-rhK (InvivoGen), respectively. An additional S136C mutation (Kabat numbering) was introduced into the rhesus macaque heavy-chain expression vector sequence to improve stability. DNA plasmids encoding the heavy and light chains were cotransfected into Expi293 cells according to the manufacturer’s protocol. Clarified supernatants were collected on day 5 posttransfection, and the antibodies were purified by protein A affinity chromatography.

### Vesatolimod pharmacokinetics.

Plasma samples were processed with a protein precipitation method using acetonitrile. The supernatant was mixed with water and injected to Sciex API-5000 LC-MS/MS system. An ACE C18 micron (50 mm by 3 mm; Advanced Chromatography Technologies, Ltd., Aberdeen, Scotland) high-pressure liquid chromatography column was used for elution and separation. The method has a lower limit of quantitation of 0.05 ng/ml and an upper limit of quantitation of 36.5 ng/ml.

### Anti-PD-1 antibody pharmacokinetics.

Nivolumab and the human/rhesus chimeric anti-PD-1 antibodies were quantified in macaque plasma using a MesoScale Discovery (MSD)-based electrochemiluminescence (ECL) assay. Briefly, a standard 96-well MSD plate (L15XA-1; MSD) was coated overnight with recombinant human PD-1 Fc chimera (1086-PD-050; R&D Systems) or anti-nivolumab Fab-fragment (AbD30255; Bio-Rad) and then blocked with MSD Blocker A (R93AA-2; MSD). Calibrators, quality controls, and unknown samples were captured by addition to the coated plates. Unbound plasma components were removed by plate washing, and the captured drug antibody was detected with a biotin-conjugated mouse anti-human IgG4 (B3648; Sigma) or anti-nivolumab antibody (AbD30258; Bio-Rad). After incubation and washing, SULFO-TAG-labeled streptavidin (R32AD-5 [MSD] or R32AD-1 [MSD]) was added to complete the final immunocomplex. After the final wash steps, the ECL signal was generated by incubation with Read Buffer T (R92TC-2; MSD) and read on an MSD Quickplex SQ120 plate reader. Unknown concentrations were back calculated to the standard curve using a five-parameter logistic regression function.

### CMV antigen-specific recall response *in vitro*.

In a CMV restimulation assay, 2.5 × 10^5^ PBMCs from a CMV-positive human donor (Cellular Technology, Ltd.) were stimulated with 0.5 μg/ml CMV pp65 pooled peptides (JPT) with 10-fold serial dilutions of nivolumab or human/rhesus chimeric anti-PD-1 antibody in 96-well flat-bottom tissue culture-treated plates. AIM-V medium (Life Technologies) supplemented with 10% heat-inactivated fetal bovine serum (FBS; HyClone/GE) and 0.1% dimethyl sulfoxide (DMSO) was used in a total volume of 200 μl/well with three to five replicates. Control wells with DMSO and antibody isotype control were plated in eight replicates. After 4 days of culture, supernatants were assayed for IFN-γ release by MSD (MesoScale Discovery).

### Rhesus macaque plasma cytokine analysis.

EDTA plasma cytokine levels were determined by Luminex assay on Luminex 200 system using a 29-plex monkey panel according to the manufacturer’s instructions (LPC0005M; Thermo Fisher).

### Plasma viral load quantification.

SIV copy number in plasma was determined by a TaqMan quantitative real-time PCR assay. Viral RNA was extracted from 200 μl of plasma using QIAamp MiniElute virus spin kit (57704; Qiagen) and amplified using the following primer/probe set: SIV Fwd, GTCTGCGTCATCTGGTGCATTC; SIV Rev, CACTAGGTGTCTCTGCACTATCTGTTTTG; and probe, 6FAM-CTTCCTCAGTGTGTTTCACTTTCTCTTCTGCG-TAMRA). All samples were amplified in triplicate in an Applied Biosystems 7500 sequence detector using the following program: 48°C for 30 min and 95°C for 10 min, followed by 40 cycles at 95°C for 15 s and 60°C for 60 s. Mean values based on extrapolations from a standard curve are reported.

### Immune cell phenotyping.

Flow cytometric analyses of frequencies of activated B cells, T cells, and monocytes were performed using EDTA anticoagulated blood predose and at 24 h postdose. The following antibody cocktail was used for B and T cell phenotyping: CD3 APC-Vio770 (130-104-236; Miltenyi), CD4 PE (550630; BD Biosciences), CD8 PerCp-Vio700 (130-097-911; Miltenyi), CD20 PE-Vio770 (130-099-733; Miltenyi), CD69 VioBlue (130-106-596; Miltenyi), CD25 BB15 (565096; BD Biosciences), and Live/Dead Aqua (1737195; Molecular Probes). The following antibody cocktail was used for monocyte phenotyping: CD14 PE-Cy7 (130-091-242; Miltenyi), CD16 APC Vio770 (130-106-765; Miltenyi), and CD169-APC (346008; BD Biosciences). Data (150,000 events) were acquired on a MACSQuant analyzer 10 and analyzed by FlowJo software.

### PD-1 receptor occupancy assay.

Cryopreserved PBMCs from the study animals at various time points were thawed and immediately stained with fixable viability dye (L34966; Thermo Fisher), anti-CD3 PerCP-Cy5.5 (552852; BD Biosciences), and nivolumab competing anti-PD1 APC/Cy7 antibody clone NAT105 (367416; BioLegend) and then fixed with BD Cytofix buffer (554655; BD Biosciences). Events were acquired on an LSR Fortessa instrument. Receptor occupancy was inferred from a decrease in anti-PD1 (NAT105) surface staining of the CD3^+^ T cell population following *in vivo* anti-PD1 chimeric antibody administration.

### SIV DNA reservoir quantification.

Cell-associated SIV DNA was quantified essentially as described previously (45, 46).

### Inducible SIV reservoir assay.

Ficoll gradient-separated, cryopreserved PBMCs from pre- and posttreatment time points were thawed and suspended in RPMI 1640 medium supplemented with 10% heat-inactivated FBS, penicillin/streptomycin, and 100 nM raltegravir. A total of 4 × 10^6^ cells were seeded per well in 12-well plates and stimulated with 5 μg/ml ConA or left untreated; each condition was tested in duplicate. After 6 days of incubation at 37°C and 5% CO_2_, cell-free supernatants were harvested, and RNA was isolated from 200-μl aliquots using a QIAmp viral minikit. SIV RNA was quantified using the TaqMan quantitative real-time PCR assay described above.

### ELISPOT analysis.

PBMCs were isolated from heparin blood using Ficoll separation and cryopreserved using a CTL immunospot freezing kit (CTLC-ABC). SIV-specific cellular immune responses were assessed using a FluoroSpot assay conducted in a 96-well plate format with 4 × 10^5^ thawed PBMCs/well stimulated for 24 h with SIV peptide pools at a 1-μg/ml final concentration, using 2 μg/ml ConA as a positive control or a plain culture medium negative control in duplicates. Spot-forming unit detection was done using an IFN/IL-2 capture kit (hT3001F; Immunospot) according to the manufacturer’s instructions and enumerated with an ImmunoSpot UV Analyzer S6 Ultimate instrument. The following SIVmac239 peptide pool reagents used in the assay were obtained through the NIH AIDS Reagent Program, Division of AIDS, NIAID: Gag peptide set (no. 6204), Env peptide set (no. 6883), Pol peptide set (no. 6443), Tat peptide set (no. 6207), Rev peptide set (no. 6448), Nef peptide set (no. 8762), Vif peptide set (no. 6205), Vpr peptide set (no. 6449), and Vpx peptide set (no. 6450). Pol and Env peptide sets were used to generate two equal-size subpools each to ensure that fewer than 200 peptides per pool were used for each stimulation.

### Statistics.

Statistical analyses were performed using Prism (version 7; GraphPad). Group statistical comparisons were done using one-way analysis of variance (ANOVA) tests with Dunnett multiple-comparison corrections, as indicated in the corresponding figure legends. *P* values of <0.05 were considered significant.

## Supplementary Material

Supplemental file 1
