# Supplementary material for: PD-1 Blockade and TLR7 Activation Lack Therapeutic Benefit in Chronic Simian Immunodeficiency Virus-Infected Macaques on Antiretroviral Therapy
Source: Antimicrob Agents Chemother. 2019 Oct 22;63(11):e01163-19. doi: 10.1128/AAC.01163-19 (PMC6811450; doi:10.1128/AAC.01163-19)
Supplement: Supplemental file 1 [file AAC.01163-19-s0001.pdf]

Supplemental Figures

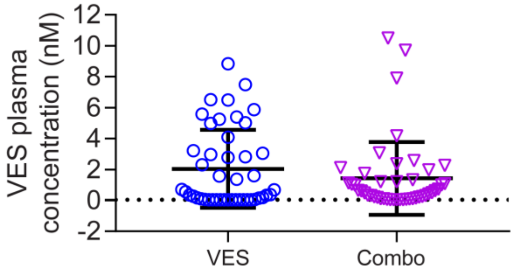

Supplemental Figure 1. VES plasma exposure. Concentration of VES in the plasma 30 min post dose assayed by LC-MS/MS. Each symbol represents one animal at one time point, with all ten time points grouped into a single column. Group mean and standard deviation are marked in black. Lower limit of quantitation (0.05 ng/ml) is marked with a dotted line.

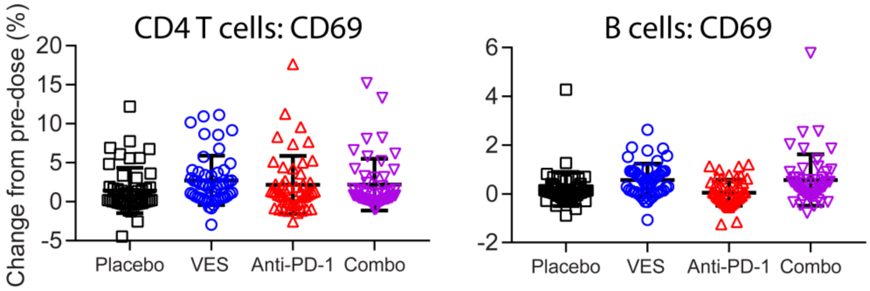

Supplemental Figure 2. CD4 T and B cell activation in response to oral dosing of VES. Percentage of CD69+ cells among peripheral CD4+ T cells (left) and B cells (right) assayed by flow cytometry and expressed as change from pre-dose to 24 hours post-dose. Each symbol represents one animal at one time point, with all the time points grouped into a single column. Group mean and standard deviation are marked in black.

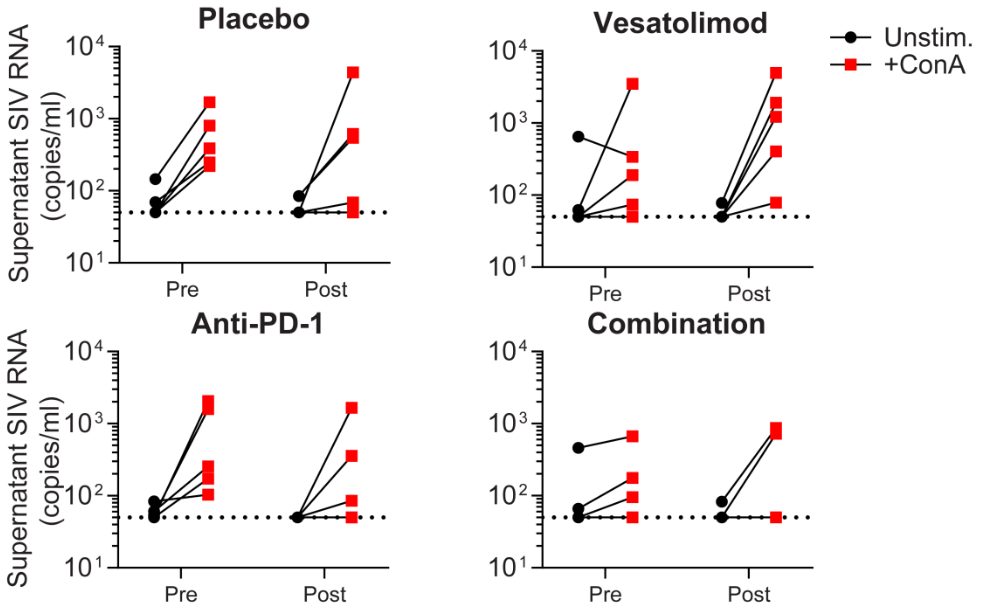

Supplemental Figure 3. ***Ex vivo*** **activation of the SIV reservoir from PBMCs.** SIV RNA quantified by qRT-PCR from the supernatants of PBMCs collected pre-therapy (week -2 relative to dose 1) and post-therapy (2 weeks post last dose) and unstimulated or stimulated with ConA for 6 days in culture.
